# Association of single nucleotide polymorphisms of miRNAs involved in the GLUT4 pathway in T2DM in a Chinese population

**DOI:** 10.1002/mgg3.907

**Published:** 2019-08-07

**Authors:** Yiping Li, Chuanyin Li, Man Yang, Li Shi, Wenyu Tao, Keyu Shen, Xianli Li, Xiaoling Wang, Ying Yang, Yufeng Yao

**Affiliations:** ^1^ Department of Endocrinology and Metabolism The Second People’s Hospital of Yunnan Province & The Fourth Affiliated Hospital of Kunming Medical University Kunming Yunnan China; ^2^ Institute of Medical Biology Chinese Academy of Medical Sciences & Peking Union Medical College Kunming Yunnan China; ^3^ Faculty of Medicine, Dentistry and Healthy Science The University of Melbourne Melbourne Vic. Australia

**Keywords:** Chinese population, GLUT4 pathway, insulin resistance, microRNA, polymorphisms, T2DM

## Abstract

**Background:**

The insulin/insulin receptor substrate (IRS)/phosphatidylinositol 3‐kinase (PI3K)/protein kinase B (Akt)/GLUT4 pathway plays a crucial role in insulin resistance and is closely associated with T2DM. Accumulating evidence indicates that miRNAs (such as miR‐135a, let‐7d, miR‐107, miR‐96, miR‐29a, miR‐23a, miR‐126, miR‐133a, and miR‐106b) influence the GLUT4 pathway.

**Methods:**

A total of 784 subjects with T2DM and 846 nondiabetic subjects were enrolled and 12 single nucleotide polymorphisms (SNPs) in miRNAs (rs10459194 in miR‐135a‐2, rs10993081 and rs7045890 in let‐7d, rs2296616 in miR‐107, rs2402959 and rs6965643 in miR‐96, rs24168 in miR‐29a, rs3745453 in miR‐23a, rs4636297 in miR‐126, rs8089787 and rs9948906 in miR‐133a‐1 and rs999885 in miR‐106b) involved in the GLUT4 pathway were genotyped using the MassArray method in a Chinese population.

**Results:**

Our data showed that the A allele of rs2402959 in miR‐96 may increase the risk of developing T2DM (*p* = .002, OR = 1.266; 95% CI: 1.089–1.471). The genotypes of rs3745453 in miR‐23a showed the difference between T2DM and control groups (*p* < .001). Moreover, for rs2402959, compared with the A/A genotype, the (G/A–G/G) genotype shows a protective effect in T2DM (*p* = .001, OR = 0.71; 95% CI: 0.58–0.87). For rs3745453, compared with the (A/A–A/G) genotype, the G/G genotype increases the risk of T2DM (*p* < .001, OR = 1.95; 95% CI: 1.38–2.77). In addition, we also found that rs4636297G/G genotype was associated with lower TC in T2DM group.

**Conclusion:**

Our results revealed that genetic variations in the miRNAs involved in the GLUT4 pathway were associated with T2DM susceptibility in a Chinese population, and these results emphasize the need to study the functional effects of these variations in the miRNAs involved in the GLUT4 pathway on the risk of developing T2DM.

## 1 INTRODUCTION

Compared with the prevalence in 2011, the prevalence of all types of diabetes, which was 10.9%, decreased slightly in China in 2013 (Wang et al., 2017; Xu et al., 2013). Type 2 diabetes mellitus (T2DM), which receives more attention as it accounts for 85%–95% of all diabetes cases, is triggered by insulin resistance in peripheral tissues.

The insulin/insulin receptor (IR)/insulin receptor substrate (IRS)/phosphatidylinositol 3‐kinase (PI3K)/protein kinase B (Akt)/glucose transport 4 (GLUT4) pathway is regarded as one of the classic insulin signaling pathways (Chakraborty, Doss, Bandyopadhyay, & Agoramoorthy, 2014; Lizcano & Alessi, 2002; Pessin & Saltiel, 2000). The binding of insulin to the IR stimulates IRS‐1 and IRS‐2 and triggers PI3K and Akt (Chakraborty et al., 2014; Lizcano & Alessi, 2002; Pessin & Saltiel, 2000). GLUT4 is widely found in insulin‐ active peripheral tissues, and its downregulation leads to insulin resistance in peripheral tissues (Aravinthan et al., 2015; Ishiki & Klip, 2005). Thus, the translocation of GLUT4 promotes the uptake of glucose. Impairment in any factors of the GLUT4 pathway leads to insulin resistance, and therefore, the development of T2DM.

MicroRNAs (miRNA or miR), which are derived from endogenous hairpin‐structured transcripts of the genome, contribute to mRNA posttranscriptional regulation. It is estimated that miRNAs regulate at least 20%–30% of all human genes (Bartel, 2004; Lewis, Burge, & Bartel, 2005). Recent studies have reported nine miRNAs and their target genes, and all of these target genes encoded proteins which are involved in the insulin/IR/IRS/PI3K/Akt/GLUT4 pathway (Agarwal, Srivastava, Srivastava, Ali, & Datta, 2013; Cao et al., 2014; Fernandez‐Twinn et al., 2014; Herrera Uribe et al., 2016; Horie et al., 2009; Jeong, Park, Yang, & Lee, 2013; Jiang et al., 2013; Ryu, Park, Ma, Zhang, & Lee, 2011; Tao et al., 2016; Yang, Jeong, Park, & Lee, 2014; Yang, Min, & Lee, 2016; Zhou, Meng, et al., 2016).

In addition, different levels of miR‐96, miR‐29a, miR‐23a, and miR‐126 are found in circulation between T2DM patients and the control group (Kong et al., 2011; Yang, Chen, et al., 2014; Zhang et al., 2013). The expression of let‐7d (Jiang et al., 2013), miR‐96 (Yang et al., 2016), miR‐29a (He, Zhu, Gupta, Chang, & Fang, 2007; Zhou, Gu, et al., 2016), miR‐126 (Fernandez‐Twinn et al., 2014), miR‐133a (Gallagher et al., 2010), and miR‐106b (Zhou, Meng, et al., 2016) are also different in skeletal muscle, adipose tissue, or liver tissue with insulin resistance and without insulin resistance. Furthermore, the expression of miR‐107 and miR‐23a changes in muscle tissue in response to loss of lean mass in obese dogs (Herrera Uribe et al., 2016). Several studies reported that single nucleotide polymorphisms (SNPs) in miRNAs have an effect on the regulation of mRNA degradation, translation, and expression of the target gene by changing the binding of miRNA recognition elements to the 3′ untranslated region of the target gene mRNA (Bartel, 2004; Guo, Ingolia, Weissman, & Bartel, 2010) and cause metabolic disease, such as T2DM (Chakraborty et al., 2014; Fernandez‐Hernando, Ramirez, Goedeke, & Suarez, 2013; Fernandez‐Valverde, Taft, & Mattick, 2011; Zhang et al., 2018).

As the insulin/IR/IRS/PI3K/Akt/GLUT4 pathway plays an important role in T2DM, we investigate the association between 12 SNPs in 9 miRNAs (rs10459194 in miR‐135a‐2, rs10993081 and rs7045890 in let‐7d, rs2296616 in miR‐107, rs2402959 and rs6965643 in miR‐96, rs24168 in miR‐29a, rs3745453 in miR‐23a, rs4636297 in miR‐126, rs8089787 and rs9948906 in miR‐133a‐1 and rs999885 in miR‐106b), which were reported to be directly or indirectly involved in the insulin/IR/IRS/PI3K/Akt/GLUT4 pathway, and T2DM in a Chinese population.

## 2 MATERIALS AND METHODS

### 2.1 Ethics statement

This study was approved by the Institutional Review Board of the Second People's Hospital of Yunnan Province. The protocol used in this investigation was in accordance with the principles expressed in the Helsinki Declaration of 1975, which was revised in 2008. All participants provided written informed consent.

### 2.2 Subjects

A total of 784 patients (495 males and 289 females) who were diagnosed with T2DM at the Second People's Hospital of Yunnan Province from February 2012 to December 2017 were recruited in this study. The diagnosis of T2DM was confirmed using the World Health Organization criteria from 1999. The NDM group included 846 subjects (503 males and 343 females) with no family history of diabetes mellitus who were recruited from individuals undergoing routine health checkups at the Second People's Hospital of Yunnan Province. Subjects with diabetes or impaired glucose tolerance were excluded from the NDM group. Subjects with a fasting plasma glucose (FPG) more than 6.1 mmol/L and/or a glycosylated hemoglobin (HbA1C) more than 6.2% further underwent an oral glucose tolerance test. In addition, subjects with hypertension or coronary heart disease were also excluded from the study. All participants (T2DM and NDM) self‐reported to be ethnically Han.

### 2.3 Laboratory measurements

Venous blood samples were collected in the morning after the subjects had fasted for 12 hr. Fasting plasma glucose (FPG) was assayed using the glucose oxidase method. The levels of total cholesterol (TC), high‐density lipoprotein cholesterol (HDL‐C), triglycerides (TG), and low‐density lipoprotein cholesterol (LDL‐C) were determined by enzymatic methods. Glycosylated hemoglobin (HbA1C) was measured by immunoturbidimetry. All laboratory measurements were performed on a HITACHI 7600‐020 Automatic Analyzer.

### 2.4 SNPs selection and genotyping

In the current study, the SNPs were selected as the candidate SNPs which are located in the promoter region, mature sequence, pri‐miRNA sequence, pre‐miRNA sequence of the miRNA genes and the 5′ and 3′ region of miRNA genes. The rs10459194 is located in the down‐stream regulatory region of miR‐135a‐2, rs10993081 and rs7045890 is located in the pri‐let‐7d region, rs2296616 is located in the pri‐miR‐107 region, rs2402959 and rs6965643 are located in the 3′ region of miR‐96, rs24168 is located in the pri‐miR‐29a region, rs3745453 is located in the 3′ region of miR‐23a, rs4636297 is located in the pri‐miR‐126 region, rs8089787 and rs9948906 are located in pri‐miR‐133a‐1 region and rs999885 is located in the promoter region of miR‐106b.

Genomic DNA was extracted from peripheral lymphocytes via the QIAamp Blood Mini Kit (Qiagen, Hilden, Germany). First, 12 SNPs in 9 miRNAs were sequenced in 50 individuals in the current population to confirm the polymorphism. Then, the 12 SNPs were genotyped using a MassArray (MassArray Analyzer 4.0, Agena, Inc). The PCR primers were designed using the AssayDesigner 3.1 (Sequenom lnc.). Four microliters PCR master mix was added into a reaction well of a 384‐well plate with 1 μl template DNA (25 ng/μl). The PCR reaction condition was performed as follows: 94°C for 5 min; followed by 45 repeat cycles of 94°C for 20 s, 56°C for 30 s and 72°C for 1 min; 72°C for 3 min as the final extension. The PCR products were treated with 2 μl shrimp alkaline phosphatase (SAP) per well to remove the dNTPs, and the reaction condition was set as: 37°C for 20 min and 85°C for 5 min. Then, 2 μl EXTEND mix was added for single base extension using the following PCR cycle condition: ①94°C for 30 s, ②94°C for 5 s, ③52°C for 5 s, ④80°C for 5 s, ⑤72°C for 3 min, and PCR stages ②–④ were repeated for 40 cycles with 5 cycles of ③ and ④per cycle. The 9 μl reaction products were purified using resin purification, and the final products were transferred to a 384‐well SpectroCHIP bioarray by a MassARRAY Nanodispenser RS1000 machine (Agena, Inc.). The MALDI‐TOF mass spectrometer (Agena, Inc.) was used to read the SpectroCHIP, and the raw genotyping data were obtained using TYPER4.0 software. To identify the accuracy of SNP genotyping by the MassArray, three SNP genotypes in each PCR product were characterized by direct sequencing on a 3,100 Genetic Analyzer (Applied Biosystems) using the BigDye Terminator v3.1 Cycle Sequencing Kit (Applied Biosystems).

### 2.5 Statistical analysis

The ages, glucose, and lipid metabolic parameters (TC, HDL‐C, TG, LDL‐C, FPG, and HbA1C) between T2DM and control groups were compared using Student's *t* test. The gender between T2DM and control groups were compared using the chi‐squared test. All polymorphic loci were tested for deviation from the Hardy–Weinberg equilibrium in the control group with a threshold of 0.05. Basic statistical analysis for allele and genotype and disease association was performed using SHEsis software (Li et al., 2009; Shi & He, 2005). Risks were estimated by the odds ratios (OR) with 95% confidence interval (95% CI). Linkage disequilibrium (LD) among these SNPs was also estimated, where the LD coefficient D was calculated using SHEsis software (Li et al., 2009; Shi & He, 2005). LD is displayed as pairwise D′, and the D′ values defined in the range [−1, 1], with a value of 1 representing perfect disequilibrium. The D′ value over 0.8 indicated the existence of different loci in the LD. The haplotypes constructed and differences in the haplotypes between the case and control groups were determined with the SHEsis software (Li et al., 2009; Shi & He, 2005). The association between each SNP and T2DM was analyzed for mode of inheritance using SNPStats (Sole, Guino, Valls, Iniesta, & Moreno, 2006). The Akaike information criterion (AIC) and the Bayesian information criterion (BIC) were used to determine the best fit model for each SNP. The glucose and lipid metabolic parameters of different genotype in each SNP were compared with one‐way ANOVA and comparisons between two groups were tested Bonferroni correction. The Student's *t* test and chi‐squared test were performed using spss 21 (Chicago, IL). Significant threshold after Bonferroni correction for multiple comparisons is *p *< .004 (0.05/12).

## 3 RESULTS

### 3.1 Subject characteristics

Table 1 lists the clinical characteristics and glucose and lipid metabolic parameters of the enrolled subjects. There were no age or gender differences between the T2DM and NDM groups, and there were no age differences between the male and female subjects of both groups (*p* > .05). However, there were significantly differences in the glucose and lipid metabolic parameters (TC, HDL‐C, TG, LDL‐C, FPG, and HbA1C) between the T2DM and NDM groups (*p* < .05) (Table 1).

**Table 1:** Clinical characteristics and glucose and lipid metabolic parameters of the subjects enrolled in the present study (Data are mean ± *SD*)

	Nondiabetic subject	Type 2 diabetes	*p*
N	846	784	
Age (years)	50.372 ± 10.238	50.619 ± 11.721	.653
Sex (M/F)	503/343	495/289	.140
Age (M; years)	49.213 ± 10.187	49.196 ± 11.857	.981
Age (F; years)	52.073 ± 10.089	53.055 ± 11.086	.248
Total cholesterol (mmol/L)	4.533 ± 0.840	4.883 ± 1.088	<.001
Triglycerides (mmol/L)	1.678 ± 1.220	2.656 ± 2.380	<.001
High‐density lipoprotein‐cholesterol (mmol/L)	1.213 ± 0.297	1.084 ± 0.276	<.001
Low‐density lipoprotein‐cholesterol (mmol/L)	2.345 ± 0.738	2.701 ± 1.022	<.001
Fasting plasma glucose (mmol/L)	4.912 ± 0.537	7.954 ± 2.557	<.001
HbA1C (%)	5.128 ± 0.373	8.911 ± 2.675	<.001

### 3.2 The sequencing results of 12 miRNA genes

Twelve miRNAs showed polymorphisms in 50 healthy individuals in the current population. The sequencing results showed that all of the 12 SNPs exhibited polymorphisms. The polymorphisms are rs10459194 in miR‐135a‐2, rs10993081 and rs7045890 in let‐7d, rs2296616 in miR‐107, rs2402959 and rs6965643 in miR‐96, rs24168 in miR‐29a, rs3745453 in miR‐23a, rs4636297 in miR‐126, rs8089787 and rs9948906 in miR‐133a‐1 and rs999885 in miR‐106b.

### 3.3 Association of the twelve SNPs with T2DM

The allele and genotype frequencies for the 12 SNPs in the miRNAs are listed in Table 2. The 12 SNPs were genotyped with a success rate between 91% and 97%. The genotype frequencies for SNPs were in HWE for the T2DM and NDM groups (*p* > .05), except for rs3745453 in the T2DM group (*p* < .05). In Table 2, 10 SNPs in 8 miRNAs (rs10459194 in miR‐135a‐2, rs10993081 and rs7045890 in let‐7d, rs2296616 in miR‐107, rs6965643 in miR‐96, rs24168 in miR‐29a, rs4636297 in miR‐126, rs8089787 and rs9948906 in miR‐133a‐1 and rs999885 in miR‐106b) showed no association with T2DM (*p* > .05). However, the allele and genotype frequency distribution of the rs2402959 in miR‐96 and rs3745453 in miR‐23a showed a different trend between the NDM and T2DM groups. The A allele of rs2402959 in miR‐96 may increase the risk of developing T2DM (*p* = .002, OR = 1.266; 95% CI: 1.089–1.471). The rs3745453 showed no significant association with T2DM after Bonferroni correction, although there was a trend association without correction (*p* = .032, OR = 0.837; 95% CI: 0.711–0.985) (Table 2).

**Table 2:** Comparison of genotypic and allelic distribution of SNPs in miRNAs mediated in GLUT4 pathway (rs10459194, rs10993081, rs7045890, rs2296616, rs2402959, rs24168, rs3745453, rs4636297, rs6965643, rs8089787, rs9948906. and rs999885) between type 2 diabetic and nondiabetic subjects

SNPs	Allele		*χ* ^2^	*p*	Odds ratio (95% CI)	Genotype			*χ* ^2^	*p*	H–W
rs10459194	C (freq)	T (freq)				C/C (freq)	C/T (freq)	T/T (freq)			
T2DM	209 (0.139)	1,291 (0.861)	0.870	.351	1.103 (0.897–1.357)	13 (0.017)	183 (0.244)	554 (0.739)	1.777	0.411	.635
NDM	206 (0.128)	1,404 (0.872)				16 (0.020)	174 (0.216)	615 (0.764)			.373
rs10993081	A (freq)	G (freq)			A/A (freq)	A/G (freq)	G/G (freq)				
T2DM	1,146 (0.786)	312 (0.214)	1.411	.235	0.899 (0.754–1.072)	458 (0.628)	230 (0.316)	41 (0.056)	2.220	0.333	.093
NDM	1,271 (0.803)	311 (0.197)				512 (0.647)	247 (0.312)	32 (0.040)			.747
rs7045890	A (freq)	G (freq)			A/A (freq)	A/G (freq)	G/G (freq)				
T2DM	1,177 (0.805)	285 (0.195)	0.207	.649	1.042 (0.871–1.247)	477 (0.653)	223 (0.305)	31 (0.042)	0.295	0.863	.448
NDM	1,236 (0.798)	312 (0.202)				495 (0.640)	246 (0.318)	33 (0.043)			.728
rs2296616	A (freq)	G (freq)			A/A (freq)	A/G (freq)	G/G (freq)				
T2DM	1,352 (0.906)	140 (0.094)	2.883	.080	0.804 (0.624–1.035)	611 (0.819)	130 (0.174)	5 (0.007)	3.222	0.200	.499
NDM	1,490 (0.923)	124 (0.077)				688 (0.853)	114 (0.141)	5 (0.006)			.906
rs2402959	A (freq)	G (freq)			A/A (freq)	A/G (freq)	G/G (freq)				
T2DM	1,018 (0.690)	458 (0.310)	9.469	.002	1.266 (1.089–1.471)	350 (0.474)	318 (0.431)	70 (0.095)	10.719	0.005	.856
NDM	1,013 (0.637)	577 (0.363)				312 (0.392)	389 (0.489)	94 (0.118)			.101
rs24168	C (freq)	T (freq)			C/C (freq)	C/T (freq)	T/T (freq)				
T2DM	902 (0.612)	572 (0.388)	0.054	.817	1.017 (0.879–1.177)	284 (0.385)	334 (0.453)	119 (0.161)	4.154	0.125	.214
NDM	947 (0.608)	611 (0.392)				277 (0.356)	393 (0.504)	109 (0.140)			.104
rs3745453	A (freq)	G (freq)			A/A (freq)	A/G (freq)	G/G (freq)				
T2DM	1,043 (0.719)	407 (0.281)	4.617	.032	0.837 (0.711–0.985)	413 (0.570)	217 (0.299)	95 (0.131)	16.481	<0.001	.001
NDM	1,167 (0.754)	381 (0.246)				448 (0.579)	271 (0.350)	55 (0.071)			.116
rs4636297	A (freq)	G (freq)			A/A (freq)	A/G (freq)	G/G (freq)				
T2DM	254 (0.166)	1,272 (0.834)	0.985	.875	0.756 (0.612–0.934)	25 (0.033)	204 (0.267)	534 (0.700)	3.671	0.160	.314
NDM	267 (0.169)	1,317 (0.831)				16 (0.020)	235 (0.297)	541 (0.683)			.099
rs6965643	A (freq)	G (freq)			A/A(freq)	A/G (freq)	G/G (freq)				
T2DM	330 (0.225)	1,136 (0.775)	0.198	.656	0.962 (0.812–1.141)	31 (0.042)	268 (0.366)	434 (0.592)	0.224	0.894	.193
NDM	359 (0.232)	1,189 (0.768)				34 (0.044)	291 (0.376)	449 (0.580)			.124
rs8089787	C (freq)	T (freq)			C/C (freq)	C/T (freq)	T/T (freq)				
T2DM	1,350 (0.906)	140 (0.094)	1.102	.750	0.961 (0.752–1.228)	612 (0.821)	126 (0.169)	7 (0.009)	0.433	0.805	.856
NDM	1,415 (0.909)	141 (0.091)				642 (0.014)	131 (0.168)	5 (0.006)			.546
rs9948906	C (freq)	T (freq)			C/C (freq)	C/T (freq)	T/T (freq)				
T2DM	1,412 (0.928)	110 (0.072)	0.488	.485	1.099 (0.843–1.432)	654 (0.859)	104 (0.137)	3 (0.004)	0.606	0.738	.598
NDM	1507 (0.921)	1,011 ((0.079)				692 (0.846)	123 (0.150)	3 (0.004)			.315
rs999885	A (freq)	G (freq)			A/A (freq)	A/G (freq)	G/G (freq)				
T2DM	1,234 (0.808)	294 (0.192)	1.415	.234	0.896 (0.748–1.074)	492 (0.644)	250 (0.327)	22 (0.029)	2.952	0.229	.143
NDM	1,330 (0.824)	284 (0.176)				549 (0.680)	232 (0.287)	26 (0.032)			.806

Note

Bonferroni correction was employed in multiple comparisons.

### 3.4 Linkage disequilibrium (LD) among SNPs in miRNA genes

According to the SNPs’ location (rs999885, rs2402959 and rs6965643 and rs24168 in chromosome 7; rs10993081, rs7045890 and rs4636297 in chromosome 9; rs8089787 and rs9948906 in chromosome 18), the LD among these SNPs was also estimated. For the SNPs in chromosome 7 (rs999885, rs2402959 and rs6965643 and rs24168), the LD test result showed that all four SNPs were not in linkage disequilibrium (D′ < 0.800). For the SNPs in chromosome 9 (rs10993081, rs7045890 and rs4636297), the LD test result showed that two SNPs (rs10993081 and rs7045890) were in linkage disequilibrium (D′ = 0.943). For the SNPs in chromosome 18 (rs8089787 and rs9948906), the LD test result showed that both SNPs in miR‐133a‐1 were in linkage disequilibrium (D′ = 0.921).

### 3.5 Association of the haplotypes of SNPs with T2DM

According to the LD test result, we did the haplotype analysis for the SNPs (rs10993081 and rs7045890 in chromosome 9; rs8089787 and rs9948906 in chromosome 18). The results showed the haplotypes were no differences between healthy and T2DM groups (data not shown).

### 3.6 Mode of inheritance analysis

To compare each inheritance model (codominant, dominant, recessive, overdominant, and log‐additive) to the most general model, the AIC and BIC were calculated to identify the inheritance model that best fit the data (Sole et al., 2006). The model with the lowest AIC and BIC value corresponds to the minimal expected entropy (Sole et al., 2006). The best fit inheritance model with the lowest AIC for rs2402959 in miR‐96 was dominant, rs3745453 in miR‐23a was recessive (Tables 3 and 4). For rs2402959, the (G/A‐G/G) genotype showed a protective effect in T2DM (*p* = .001, OR = 0.71; 95% CI: 0.58–0.87) in the dominant inheritance model. For rs3745453, the G/G genotype showed a risk effect in T2DM (*p* < .001, OR = 1.95; 95% CI: 1.38–2.77) in the recessive inheritance model. The other SNPs showed no differences between the NDM and T2DM groups (data not shown).

**Table 3:** Different inheritance models analysis of the SNP rs2402959 in miR‐96 between the NDM and T2DM group

Model	Genotype	NDM (freq)	T2DM (freq)	Odds ratio (95% CI)	*p*	AIC	BIC
Codominant	A/A	312 (0.392)	350 (0.474)	1.00			
	G/A	389 (0.489)	318 (0.431)	0.72 (0.59–0.90)	.004	2,117.9	2,144.6
	G/G	94 (0.118)	70 (0.095)	0.66 (0.47–0.93)			
Dominant	A/A	312 (0.392)	350 (0.474)	1.00	.001	2,116.2	2,137.6
	G/A–G/G	483 (0.608)	388 (0.526)	0.71 (0.58–0.87)			
Recessive	A/A–G/A	701 (0.882)	668 (0.905)	1.00	.140	2,124.8	2,146.1
	G/G	94 (0.118)	70 (0.095)	0.78 (0.56–1.08)			
Overdominant	A/A–G/G	406 (0.511)	420 (0.569)	1.00	.019	2,121.5	2,142.9
	G/A	389 (0.489)	318 (0.431)	0.79 (0.64–0.96)			
Log‐additive	—	—	—	0.78 (0.67–0.91)	.002	2,116.9	2,138.3

Note

Bonferroni correction was employed in multiple comparisons.

**Table 4:** Different inheritance models analysis of the SNP rs3745453 in miR‐23a between the NDM and T2DM group

Model	Genotype	NDM (freq)	T2DM (freq)	Odds ratio (95% CI)	*p*	AIC	BIC
Codominant	A/A	448 (0.579)	413 (0.570)	1.00	<.001	2,067.3	2,093.8
	A/G	271 (0.350)	217 (0.299)	0.87 (0.69–1.09)			
	G/G	55 (0.071)	95 (0.131)	1.86 (1.30–2.66)			
Dominant	A/A	448 (0.579)	413 (0.570)	1.00	.740	2,081.3	2,102.6
	A/G–G/G	326 (0.421)	312 (0.430)	1.04 (0.84–1.27)			
Recessive	A/A–A/G	719 (0.929)	630 (0.869)	1.00	<.001	2,066.8	2,088.1
	G/G	55 (0.071)	95 (0.131)	1.95 (1.38–2.77)			
Overdominant	A/A–G/G	503 (0.650)	508 (0.701)	1.00	.037	2,077.1	2,098.3
	A/G	271 (0.350)	217 (0.299)	0.79 (0.64–0.99)			
Log‐additive	—	—	—	1.16 (1.00–1.35)	.051	2,077.6	2,098.9

Note

Bonferroni correction was employed in multiple comparisons.

### 3.7 The association of genotype with metabolic parameters analysis

In NDM group, no significant association of genotype of 12 SNPs in 9 miRNAs (rs10459194 in miR‐135a‐2, rs10993081 and rs7045890 in let‐7d, rs2296616 in miR‐107, rs2402959 and rs6965643 in miR‐96, rs24168 in miR‐29a, rs3745453 in miR‐23a, rs4636297 in miR‐126, rs8089787 and rs9948906 in miR‐133a‐1 and rs999885 in miR‐106b) with glucose and lipid metabolic parameters, including FPG, TC, HDL‐C, TG, LDL‐C, and HbA1C was observed in the current study (data not shown).

Otherwise, in T2DM group, there were significant statistic differences for TC among rs4636297 A/A, A/G and G/G genotype (*p* = .005). Furthermore, when comparing with A/A genotype, G/G genotype was associated with lower TC after Bonferroni correction (*p* = .011, OR = 0.645; 95% CI: 0.116–1.174). However, there were no significant associations of genotypes of other 11 SNPs with glucose and lipid metabolic parameters and no significant associations of genotype of rs4636297 with other metabolic characteristics except TC (Table 5).

**Table 5:** The genotype of rs4636297 in miR‐126 with total cholesterol (TC) in T2DM group

Genotype	*N*	TC (mmol/L)	*p*	*p* (A/A vs. A/G) Odds ratio (95% CI)	*p* (A/G vs. G/G) Odds ratio (95% CI)	*p* (A/A vs. G/G) Odds ratio (95% CI)
A/A	25	5.452 + 1.111		.109 0.479 (−0.069 to 1.028)	.200 0.166 (−0.051 to 0.382)	.011 0.645 (0.116–1.174)
A/G	197	4.973 + 1.043	.005			
G/G	521	4.808 + 1.087				

Note

Bonferroni correction was employed in multiple comparisons.

## 4 DISCUSSION

The association of SNPs in miRNAs was widely studied in metabolic diseases, autoimmune diseases, and cancers. Until this study, few studies focused on the SNPs in a group of miRNAs involved in a disease‐related pathway and examined the association between these SNPs and T2DM. In the current study, we investigated the association between 12 SNPs in 9 miRNAs, which were reported to be involved in the insulin/IR/IRS/PI3K/Akt/GLUT4 pathway, and T2DM in a Chinese population. Our results showed that the SNPs rs2402959 in miR‐96 and rs3745453 in miR‐23a were identified as being associated with T2DM in this Chinese population.

In 2016, Yang et al reported that miR‐96 played a crucial role in the development of hepatic insulin resistance in saturated fatty acids‐induced obesity. miR‐96 directly downregulated INSR and IRS‐1 expression by binding to their 3' UTRs in hepatocytes (Yang et al., 2016). Moreover, the level of miR‐96 was upregulated in the hepatocytes of mice with insulin resistance (Yang et al., 2016) and downregulated in the serum of T2DM patients (Yang, Chen, et al., 2014). However, until now, no studies reported the association of SNPs located in miR‐96 with T2DM. In the current study, we found that the A allele of rs2402959 in miR‐96 may increase the risk of developing T2DM (*p* = .002, OR = 1.266; 95% CI: 1.089–1.471). The contribution of the SNP rs2402959 in the A allele to the expression of miR‐96 has not been identified. It is possible that the risk allele is in linkage disequilibrium with the other functional variants, although it is speculated to have no effects on the biogenesis and function of miR‐96 (Sanchez‐Mora et al., 2013). In 2013, Sanchez‐Mora *et al* reported that the T allele of rs2402959 and the A allele of rs6965643 in miR‐96 were risk factors for children with attention deficit‐hyperactivity disorder but without substance use disorders (*p* = .047 and .029 respectively) (Sanchez‐Mora et al., 2013). However, rs6965643 in miR‐96 was not found to associate with T2DM in the current study. One reason for the difference between the Sanchez‐Mora *et al* study and our study could be the different genetic background between the European and Asian population. For rs6965643, the A allele was the minor allele, and the frequency was approximately 20%–27% in the Asian population. However, the G allele was the minor allele with a frequency of 20%–28% in the European population (http://asia.ensembl.org/Homo_sapiens/Variation/Explore?db=core;r=7:129767224-129768224;v=rs6965643;vdb=variation;vf=416120248). Moreover, there was no linkage disequilibrium observed between rs2402959 and rs6965643 in the current population (D′ = 0.268 and *r* ^2^ = .011).

In 2016, Herrera Uribe *et al* reported that miR‐23a involved in the GLUT4 pathway might directly or indirectly regulate the GLUT4 gene as a decreased expression of miR‐23a accompanied the downregulation of the GLUT4 gene in muscle tissue in response to the loss of lean mass in obese dogs (Herrera Uribe et al., 2016). Furthermore, Yang *et al* found that miR‐23a expression was significantly decreased not only in the serum of T2DM and prediabetes patients compared with that of normal glucose tolerant patients but also in the serum of T2DM patients compared with prediabetes patients (Yang, Chen, et al., 2014). These results indicated that downregulation of miR‐23a expression was a risk factor for T2DM (Yang, Chen, et al., 2014). In the current study, we found that the rs3745453 showed a trend association with T2DM (*p* = .032, OR = 0.837; 95% CI: 0.711–0.985); however, the rs3745453 showed no associated with T2DM after Bonferroni correction. In 2013, Ridolfi *et al* reported that the C allele of rs3745453 in miR‐23a could be a risk factor for multiple sclerosis (Ridolfi et al., 2013). Moreover, these authors found that the C allele of rs3745453 in miR‐23a could be associated with reduced miR‐23a levels in serum in multiple sclerosis patients compared with control subjects. The C allele of rs3745453 in miR‐23a could be associated with the downregulation of miR‐23a. Thus, rs3745453 A allele in miR‐23a could be associated with the higher miR‐23a level, which is a protective factor in the development of T2DM.

miR‐29a, on the one hand, reduced GLUT4 expression by targeting the secreted protein acidic rich in cysteine (SPARC) protein in 3T3‐L1 adipocytes, thus negatively regulating glucose metabolism (Song, Ding, Zhang, & Wang, 2018). On the other hand, the overexpression of miRNA‐29a impaired the insulin‐induced glucose uptake by indirectly acting on Akt, leading to insulin resistance in 3T3‐L1 adipocytes (He et al., 2007). The role of miR‐29a in glucose metabolism was observed not only in adipocytes but also in skeletal muscle cells, as the overexpression of miR‐29a led to insulin resistance by inhibiting the proliferator‐activated receptor δ(PPARδ), and peroxisome proliferator‐activated receptor‐γ coactivator‐1α (PGC‐1α) signals and GLUT4, which are responsible for insulin‐stimulated glucose uptake (Zhou, Gu, et al., 2016). Moreover, miR‐29a was upregulated in sera in newly diagnosed T2DM compared with the pre‐diabetes (Kong et al., 2011). However, we showed that the miR‐29a rs24168 was not associated with T2DM in the present study.

The let‐7d miRNA was reported to increase glucose uptake via an Akt‐dependent mechanism by repressing IL‐13 in skeletal muscle (Jiang et al., 2013). Moreover, Jiang *et al* reported a relatively higher let‐7d expression in cultured myotubes in T2DM subjects than that in NGT (normal glucose‐tolerant) subjects (*p* = .08) (Jiang et al., 2013). In 2015, Yan *et al* reported that although there were no associations of the let‐7d rs10993081 allele in the dominant and recessive inheritance models with T2DM, the A/G genotype of rs10993081 in let‐7d was associated with an increased risk of metabolic syndromes compared with AA genotypes (*p* = .006; OR = 1.42, 95% CI: 1.11–1.83) (Yan et al., 2015). In the current study, we also did not find that rs10993081 and rs7045890 in let‐7d were associated with T2DM. One reason we did not find an association of both rs10993081 and rs7045890 in let‐7d in patients with T2DM could be that metabolic syndrome includes abdominal obesity, dyslipidemia, and elevated blood pressure in addition to high blood glucose, and T2DM is only one of these metabolic diseases.

In 2014, Fernandez‐Twinn *et al* reported that miR‐126, which was overexpressed in the adipose tissue of mice with maternal obesity, played a posttranscriptional downregulation role for IRS‐1 in adipocytes (Fernandez‐Twinn et al., 2014). However, miR‐126 expression was significantly downregulated in the plasma of T2DM patients (Zhang et al., 2013). In 2015, McAuley *et al* identified that the miR‐126 rs4636297 A allele, which increased the expression of miR‐126 by facilitating the progression of pri‐miRNA to mature miRNA, was associated with sight threatening diabetic retinopathy (McAuley et al., 2015). However, in the current study, we did not find that rs4636297 in miR‐126 was associated with T2DM. One of the reasons could be that miR‐126 is involved different pathways in T2DM and diabetic retinopathy. The miR‐126 may contribute to diabetic retinopathy by affecting vascular endothelial growth factor (Fish et al., 2008), which plays an important role in the neovascularisation of diabetic retinopathy. While for T2DM, miR‐126 contributes to insulin resistance by affecting IRS‐1 in the GLUT4 pathway (Fernandez‐Twinn et al., 2014). In addition, Ciccacci *et al* observed a differing role for miR‐27a rs895819 in diabetes and in diabetic complications (Ciccacci et al., 2013, 2014). Moreover, in the current study, rs4636297G/G genotype was identified to be associated with lower TC in T2DM group. Recent study showed that rs4636297G allele led to the lower mature miR‐126 expression (Harnprasopwat et al., 2010) and the lower mature miR‐126 was a risk factor of T2DM (Zhang et al., 2013), which indicated that the rs4636297G/G genotype should be a risk genotype of T2DM. However, we did not observe the association between rs4636297G/G and T2DM in the current study. The reason of the lack association might be that favourable effects of the SNP on lipid metabolism protect from the development of T2DM. The function investigation of rs4636297 in miR‐126 should be studied in the future.

## 5 CONCLUSIONS

As the SNPs in miRNAs affect the regulation of target genes, and miRNAs play an important role in T2DM through affecting the GLUT4 pathway on insulin resistance, our study provided a novel perspective to explore the associations of twelve SNPs located in 9 miRNAs involved in the GLUT4 pathway with T2DM. Our results showed that rs2402959 in miR‐96 and rs3745453 in miR‐23a showed an association with T2DM in a Chinese population. In addition, rs4636297G/G genotype was associated with lower TC in T2DM group. In the future, the functional effects of these variations in miRNAs on the target genes of the GLUT4 pathway, insulin resistance and T2DM need to be further investigated.

## CONFLICT OF INTEREST

None declared.
